# Metropolitan Hospital Sunday Fund

**Published:** 1896-08-01

**Authors:** 


### The Institutional Workshop.

### METROPOLITAN HOSPITAL SUNDAY FUND.

The annual meeting of the Council of the Metropolitan Hospital Sunday Fund was held at the Mansion House, on Tuesday, 28th ult. Sir Sydney H. Waterlow, Bart. (vice-president), in the absence of the Lord Mayor, took the chair.

The Chairman, in moving the adoption of the report of the Committee of Distribution (which we give elsewhere), and the payment of the awards as recommended by them, said that although the amount of the collection in 1896 (£44,684) was not so large as that of the previous year (which was very much larger than usual owing to circumstances well known to all present), still on the whole they might well be satisfied with it, especially when they considered that during the past few months an attempt had been made to raise an endowment fund for one of the largest metropolitan hospitals. After expressing his thanks to Sir Savile Crossley, Bart., and other donors who for years had contributed to the Fund, and to the Rev. Canon Fleming, at whose church the largest collection had once more been made, the Chairman said that year after year the work of the Distribution Committee increased, and in no year had so many meetings been held as last year.

Sir Richmond Cotton asked whether the case of St. Thomas's Hospital had been taken into consideration by the Distribution Committee. St. Thomas's was purely a City hospital, and was really very much in want of funds.

The Chairman said that St. Thomas's Hospital had entered into communication with the committee, but after some correspondence had passed it withdrew its application. As far as he was able to learn, its objection was to the preparation of its accounts on the uniform system, and the auditing of them by a public accountant.

Captain Arthur Palliser seconded the adoption of the report.

Dr. Glover said that before the resolution was put he had a complaint to make. At a meeting in January he brought under the notice of the Council the great and growing proportions of the out-patient department of London hospitals. That matter was referred to the Distribution Committee, and he was disappointed to find that the subject had been absolutely ignored in their report. A special meeting of the committee, to which he and other gentlemen were invited, had been held to consider the matter, and he had left that meeting with the understanding that a summary of the facts regarding the out-patient departments of the London hospitals, showing the numbers of and the total expenditure on out-patients, and the average expenditure on each out-patient at the different hospitals, was to be prepared, so as to enable the Council to understand what a large expenditure these departments involved. To his great surprise not a word on the subject was said in the report. Not only was he disappointed at this, but he thought the Council and the public would be disappointed. There was a very strong feeling outside that the out-patient department was open to criticism, and he submitted that the Council of that Fund, whose business it was to make a great collection for hospitals, should consider it part of their duty to express their opinion on what seemed to be a great and growing evil. The very hospitals themselves would be disappointed that the Council had not moved in the matter, for they admitted that this question was the weak point in their administration, and he believed they would be thankful for the moral support which would enable them to exercise the care requisite to reduce in some way the great proportions of this evil. He disclaimed any unfriendly feeling to hospitals, and he recognised the educational and charitable use of the out-patient departments, but even from the charitable and educational point of view, it had been admitted that the numbers at present attending were too great. Hospitals were, as a matter of fact, becoming huge dispensaries, and were undertaking the care of patients who should be attended by the provident dispensaries, or by the humbler classes of general practitioners. Eighty-two of the hospitals which received grants from that Fund spent on their out-patient departments £94,604, and each out-patient cost on an average 3s. 5¾d. He characterised the treatment the Council had received at the hands of the Distribution Committee as unsympathetic and slightly disrespectful. If the subject had been faced their collections would be increased, for the public would be more inclined to put its money into the hands of the Council if they were not so timid about telling the hospitals what were the weak points in their administration. A reasonable reduction in the out-patient department of all the hospitals would result in a saving of from £30,000 to £40,000 a year, and that with slightly increased collections by the Fund would mean the £100,000 a year which they were told the London hospitals stood in need of.

Mr. Henry C. Burdett said it was only fair to point out that when the subject was previously discussed the opinion of the Council, as represented by the speakers who joined in the discussion, was that, on the whole, the question had better be left alone. The Distribution Committee was the neutral side of the Hospital Sunday Fund, and it was, on the whole, thought that it was not their duty to bring forward any recommendation or suggestion on a subject on which the opinion of the Council, so far as interference was concerned, was equal, or, perhaps, more inclined to be negative. He agreed with Dr. Glover that the out-patient question, as represented by the amount of relief given by the metropolitan hospitals to-day, was a scandal. It was more—it was a danger to the whole population, because it undermined the principle of thrift, which should animate every citizen, and it perpetrated a gross injustice in the sense that the teachers and leaders of the medical profession, who were responsible for the present position, allowed it to go on to the injury of the humbler members of the profession, who had attended the schools and received their education from the very hospitals which were competing with them. The worst side of it was that every poor man in this City had the right to have medical attendance at his own door, and he could not hope to get this if the hospitals admitted such a large and increasing number of all sorts and conditions of men and women without sufficient enquiry, that it was almost impossible for the medical man who had to depend for his living upon small fees, to reside in the most crowded parts of the metropolis. Admitting all this, however, he ventured to think that the solution of the difficulty could not be looked for from that Council, which, after all, was a collecting body more than a criticising body. The solution must come from the leaders of the medical profession themselves and from the body of general practitioners who suffered from the growth of the out-patient department. If the general practitioners realised their position, he could not but believe that they would go to their own schools and to the consultants (who after all depended upon the general practitioner for their living), and represent that the out-patient system as at present maintained was likely to end in the injury of both parties. Then representations could be made to the hospital managers. It was the duty of the medical profession to look this matter in the face and to deal with it on some principle which would reduce the evil and secure the proper interests of the medical man and the public. Until this had been done he hoped the Council of the Hospital Sunday Fund would be content to do its work of collecting money without indulging in criticisms which must necessarily be directed to points which it could not follow up by enforcing reforms of a sufficiently drastic nature. The Council would be well-advised not to take any steps in the direction of attempting to interfere with the present system of out-patient relief. The managers of hospitals themselves could not effectually interfere until they had a request put forward in a practical form bv their medical staff, who had it in their power to remedy the defect.

The Chairman explained that the reason why the Distribution Committee had not mentioned the matter in their report was that there was so much diversity of opinion on the subject, they did not see their way to recommend any course to the Council. He did not agree with Dr. Glover's statistics, as there was nothing more difficult than to discover, where separate accounts for in and out patients were not kept, what the treatment of the out-patients really cost. The returns, therefore, which the hospitals themselves made were most unreliable and ought not to form any part of statistics on which action was to be taken.

The report was then adopted.

Mr. Bryant proposed—

"That the cordial thanks of the Council be and are hereby given to Sir Sydney H. Waterlow, Bart. (chairman), and to the other members of the Committee of Distribution, for the considerable care and attention bestowed in the preparation of the awards they have recommended, and for the very efficient report of their proceedings."

Dr. Hare seconded, and the motion was carried.

Rev. C. J. Ridgway, in proposing—

"That the thanks of the Council be and are hereby given to the editors of newspapers who have pleaded the needs of hospitals, and advocated the cause of this Fund, with a view to increasing this year's collection,"

said that The Hospital and *The Lancet*, who prepared statistics for distribution on Hospital Sunday, were entitled to special thanks. It was from want of using the facts provided by these newspapers on the part of many of his brother clergymen that larger collections were not obtained in many of the churches of the metropolis.

Mr. Walter R. Tidd seconded, and the resolution was passed unanimously.

Sir Richmond Cotton proposed—

"That the cordial thanks of the Council are now given to the Right Honourable Sir Walter Wilkin, Bart., Lord Mayor, who, as president and treasurer, has devoted his valuable time and made special efforts to promote the growth of the Fund."

Mr. Burdett, in seconding, mentioned that, at the Lord Mayor's suggestion, the experiment of selecting a number of churches at which special exertions should be made had been tried this year, with the result that at these churches, on an average, £30 more per congregation had been received.

The motion having been carried unanimously,

A vote of thanks to the Chairman was passed on the motion of Mr. Joseph Fry, seconded by Dr. Glover, and

The proceedings terminated.

